# Ectopic Expression of the RING Domain of the Arabidopsis PEROXIN2 Protein Partially Suppresses the Phenotype of the Photomorphogenic Mutant *De-Etiolated1*

**DOI:** 10.1371/journal.pone.0108473

**Published:** 2014-09-23

**Authors:** Mintu Desai, Navneet Kaur, Jianping Hu

**Affiliations:** 1 Michigan State University-Department of Energy Plant Research Laboratory, Michigan State University, East Lansing, Michigan, United States of America; 2 Plant Biology Department, Michigan State University, East Lansing, Michigan, United States of America; University of South Florida College of Medicine, United States of America

## Abstract

The Arabidopsis CONSTITUTIVE PHOTOMORPHOGENIC/DE-ETIOLATED 1/FUSCA (COP/DET1/FUS) proteins repress photomorphogenesis by degrading positive regulators of photomorphogenesis, such as the transcription factor LONG HYPOCOTYL5 (HY5). The gain-of-function mutant *ted3*, which partially suppresses the *det1* mutant, contains a missense mutation of a Val-to-Met substitution before the C-terminal RING finger domain of the peroxisomal membrane protein PEROXIN2 (PEX2). We hypothesized that a truncated PEX2 protein, which only contains the C-terminal RING domain, is initiated by the *ted3* mutation and by-passes the function of DET1 in the nucleus. Although we have not been able to detect this hypothetic peptide *in vivo*, we show in this study that, when fused with a fluorescent protein and overexpressed, the PEX2 RING domain can localize to the nucleus, where it is able to interact with HY5, and PEX2 RING domain overexpression in *det1* also partially suppresses the *det1* phenotype. Compared with *det1*, *ted3 det1* plants have significantly decreased levels of the HY5 protein and the expression of most of the analyzed HY5 target genes is altered to levels comparable to those in *hy5*. We conclude that compromised activity of HY5 may have been mainly responsible for the partial reversal of the *det1* phenotype in *ted3 det1*. Our data support the notion that, when appropriately localized, some RING finger domains may be able to achieve neomorphic effects in the cell.

## Introduction

In response to the changing light regime, seedlings of higher plants undergo two drastically different programs. Skotomorphogenesis (etiolation) takes place in the dark, during which seedlings develop a long hypocotyl and hooked/undeveloped cotyledons. When exposed to light, seedlings go through photomorphogenesis (de-etiolation), where hypocotyl growth is inhibited, cotyledons open, chloroplasts develop, and genes involved in photosynthesis and light-regulated development are expressed. Light signals are transduced from photoreceptors, early signaling factors, central integrators, to downstream effectors, resulting in changed expression of hundreds of genes [1], [2], [3].

As central regulators of photomorphogenesis, CONSTITUTIVE PHOTOMORPHOGENIC/DE-ETIOLATED 1/FUSCA (COP/DET1/FUS) proteins comprise three distinct protein complexes in a ubiquitin (Ub)-proteasome system. This system targets key positive regulators of light response, such as the photoreceptor phytochrome A (phyA) and transcription factors Long Hypocotyl5 (HY5)/HY5 Homolog (HYH), Long Hypocotyl in Far-Red1 (HFR1), and Long After Far-Red Light1 (LAF1), for degradation. COP1 is a RING finger-containing E3 ligase that acts as a central component of a CULLIN4 (CUL4)-based E3 complex. Besides being a chromatin regulator to repress gene expression, DET1 is part of another CUL4-based E3 complex that functions to enhance the activity of the COP1 complex with the help of the COP9 signalosome (CSN) [4], [5]. The bZIP transcription factor HY5 is a master regulator of photomorphogenesis that controls the expression of a repertoire of light-response genes [6]. In the dark, COP1 transits from cytoplasm to the nucleus, where it interacts with HY5 and mediates its ubiqutination by the concerted activity of COP/DET1/FUS protein complexes, resulting in significant reduction of HY5 abundance due to protein degradation by the 26S proteasome [7]. As such, dark-grown loss-of-function mutants of most *COP/DET1/FUS* genes show developmental patterns akin to that in light-grown wild-type seedlings (i.e. de-etiolated), whereas seedlings of photomorphogenesis-promoting factors such as HY5 often have long hypocotyls in the light [4].

Light regulates the development and function of subcellular organelles as well. In addition to its well-known impact on chloroplasts, light has also been linked to peroxisomes, essential eukaryotic organelles that mediate a variety of metabolic processes, such as photorespiration, fatty acid β–oxidation, and biosynthesis and metabolism of hormones in plants [8], [9]. Light up-regulates the expression of genes encoding enzymes involved in photorespiration – a process that accompanies photosynthesis, while it represses genes involved in fatty acid β–oxidation and the glyoxylate cycle – processes that provide energy to seedling establishment before photosynthesis begins [10]. Light also promotes the proliferation of peroxisomes in Arabidopsis seedlings through phyA and the bZIP transcription factor HYH, the latter of which directly binds to the promoter and presumably activates the expression of the peroxisome proliferation factor gene *PEX11b* [11], [12]. This is consistent with the idea that during photomorphogenesis, an increase in peroxisomal population takes place besides the activation of the expression of photorespiratory genes and the import of their products into the peroxisome.

Before the discovery of DET1 as part of the protein complexes that degrade positive regulators of photomorphogenesis, the *det1-1* allele was used as the background to isolate extragenic suppressors to investigate the function of the DET1 protein [13]. One partial suppressor, *ted3* (for reversal of *det*), turned out to carry a gain-of-function mutation in the peroxisome biogenesis factor *PEROXIN2* (*PEX2)* [14]. PEX2 is a conserved RING finger domain-containing peroxisomal membrane protein involved in peroxisomal protein import in diverse species. PEX2 or its RING domain possesses E3 ubiquitin ligase activity in yeast *Saccharomyces cerevisiae* [15], mammals [16], and Arabidopsis [17].

Multiple models have been proposed to explain the partial suppression of *det1* by *ted3* [10]. One model postulated that DET1 is a key positive regulator of peroxisomal functions and that *ted3* possesses enhanced peroxisomal activities to suppress *det1-1*. Some of the phenotypes in *det1-1* are similar to those in peroxisomal β-oxidation mutants, such as sugar-dependent seedling establishment and partial resistance to indole-3-butyric acid (IBA), a protoauxin that is converted to the bioactive auxin indole-3-acetic acid (IAA) by β–oxidation [14]. However, viable loss-of-function peroxisomal mutants do not have opened cotyledons like *det1* despite having shorter hypocotyls on media without sucrose, arguing that peroxisomes do not play a major role in photomorphogenic development but rather represent one of the many downstream branches in DET1’s regulatory network in growth and development. In addition, DET1 represses photomorphogenesis yet light activates photorespiration and peroxisomal proliferation, suggesting that DET1 is not a primary regulator of general peroxisomal function.

A second hypothesis favored the scenario that *ted3* encodes a gain-of-function product, which bypasses the function of DET1 in photomorphogenesis. The *ted3* mutation contains a G-to-A transition that leads to a Val-to-Met substitution one amino acid upstream from the first Cys of the C-terminal RING finger domain [14]. It is conceivable that in *ted3 det1*, this new Met may initiate the translation of a cryptic peptide that comprises the RING finger domain. Alternatively, changing from Val to Met may increase the accessibility of the protein to cytoplasmic proteases, which cleave off the cytosolically exposed RING domain of PEX2. This RING domain from PEX2, which has been shown to contain E3 ubiquitin ligase activity *in vitro* [17], may be mobilized to the nucleus because of its small size (∼6 kDa) and substitute for the function of the COP1-DET1 E3 ligase complexes in degrading some of the positive regulators of photomorphogenesis. We have not been able to detect this small peptide *in vivo*. However, in this study we have provided evidence that the RING domain of PEX2 when overexpressed is able to partially rescue the *det1* phenotype. PEX2’s RING domain can enter the nucleus, where it interacts with the transcription factor HY5 and presumably reduces its function. We postulate that this alteration of HY5 activity may largely account for the partial reversal of the *det1* phenotypes in the *ted3 det1* dominant mutant during photomorphogenesis.

## Materials and Methods

### Plant growth, light conditions and genetic crosses

The wild-type Arabidopsis plants used in this study were from the Columbia-0 (Col-0) ecotype. *hy5-1*, *cop1-4*, *det1-1* and *ted3 det1* were in the Col-0 background. These mutants were confirmed by their respective dark-grown phenotypes, and genotyped by PCR analysis to ensure their homozygosity. Seeds were surface sterilized with 20% Clorox and 0.025% Triton X-100, washed 5 times with sterile water. To measure hypocotyl length, sterilized seeds were plated on 0.5X MS medium supplemented with 0.5% sucrose and solidified with 0.6% phytagar, stratified at 4°C for 3d, exposed to white light (100 µm m^−2^s^−1^) for 1 h to induce synchronous germination, and returned to the darkness for 4d at 22°C. Hypocotyl lengths of >30 seedlings from each genotype were measured using ImageJ software (http://imagej.nih.gov/ij/). Three biological replicates were undertaken. After having acquired their first true leaves, the seedlings were transferred to soil and grown in growth chambers with 100 µm m^−2^s^−1^ white light, 16/8 h photoperiod, and at 22°C.

### Confocal laser scanning microscopy (CLSM) and epifluorecence microscopy

Plant tissues (as indicated in the text) were incubated with DAPI (Invitrogen, Carlsbad, CA) at 300 nM concentration in 1X PBS at room temperature, covered with aluminum foil for 15 min followed by 3–4 washes to remove excess stain, and directly mounted in distilled water to be analyzed by CLSM (Zeiss LSM 510 META). A 488-nm, 514-nm argon ion laser and 401-nm diode were used for excitation; emission filters of 505–530 nm, 520–555 nm band-pass and 433-nm long-pass were used for GFP, YFP and DAPI respectively. Images were acquired at 63X with oil. Epifluorescence microscopy was performed with an Axio Imager M1 microscope (Carl Zeiss) for visualization of the BiFC between HY5-YFPct and YFPnt-PEX2RF proteins (excitation 500±12 nm; emission 542±13.5 nm).

### RT-PCR analyses

Total RNA was isolated from 4d dark-grown seedlings using SV total RNA isolation system kit (Promega, Madison, WI). For RT-PCR analysis, 2 µg total RNA was reverse-transcribed with the Omniscript RT kit (Qiagen, Valencia, CA). PEX2 RF-specific primers FW (5′-GTGACTTGCCCTATTTGC-3′) and RE (5′-TCATTTGCCACTTGAAAC-3′) were used to amplify a 0.1-kb product that covered the entire C-terminal end containing the RF domain from *PEX2* cDNA. *UBQ10*-FW (5′-TCAATTCTCTCTACCGTGATCAAGATGCA-3′) and *UBQ10*-*RE* (5′-GGTGTCAGAACTCTCCACCTCAAGAGTA-3′) from the *UBQ10* gene (At4g05320) were used to amplify a product of ∼320 bp that served as an internal control. For *PEX2* RF domain and *UBQ10* amplification, PCR was performed with the following conditions: 94°C for 2 min, 30 cycles of 94°C for 30 s, 57°C for 30 s, 72°C for 30 s, and a final extension at 72°C for 4 min.

### Quantitative real-time PCR

For transcript analysis, whole Arabidopsis seedlings grown under constant light at 22°C on 0.5X MS media plates were used. Harvested seedling samples were frozen in liquid N_2_ and total RNA was extracted using RNeasy plant mini kits (Qiagen, Valencia, CA) followed by treatment with DNase I (Qiagen, Valencia, CA) according to manufacturer’s instructions. Synthesis of cDNA was performed with the Omniscript Reverse Transcription system (Qiagen, Valencia, CA) using random primers with 0.1 µg of total RNA in a 20 µl volume RT reaction, and incubated for 1 hr at 42°C. The RT reaction mixture was diluted 10-fold and 1 µl was used as a template in 10-µl PCR reaction, using the Applied Biosystems FAST7500 Real-Time PCR systems in fast mode and FAST SYBR GREEN PCR Master Mix (Applied Biosystems, Foster City, CA), following the manufacturer’s protocol. Cycling conditions were as follows: 8 min at 95°C, 40 cycles of 10 s at 95°C, 30 s at 58°C, and 30 s at 72°C, followed by a 60 to 95°C dissociation protocol. The primers for transcript analysis were designed by the primer express software (Applied Biosystems, Foster City, CA) and are listed in Table S1. All reactions were performed in triplicate and the products were checked by melting curve analysis. Sequence of the PCR products had been confirmed. The transcript level was measured by normalizing the level with that of the *UBQ10 as* reference transcript. Each experiment was repeated at least 2 times. The values are average of three biological replicates which yielded consistent results.

### Plasmid construction

For all the plasmid construction, PFU turbo (Invitrogen, Carlsbad, CA) was used. PEX2RF was amplified from pCHF3-PEX2 [14] by PCR with primers introducing a *Kpn*-I site at the 5′ of FW and *Sac*-I at the 3′ end of RE. The amplified PCR product was confirmed by sequencing and cloned into pCHF3:GFP [14] to generate pHU006 and into a pCAMBIA vector (Cambia, Canberra) to generate pHU007. By floral dipping [18], pHU006 was transformed into Col-0 for PEX2RF-GFP the localization study, using Hygromycin for selection, and pHU007 was transformed into *det1-1* for the complementation study, using Kanamycin for selection. T2 transgenic plants were used for further analyses.

To express the PEX2RF protein for antibody generation, specific oligonucleotides were synthesized and cloned at *Nco* I and *Xho* I sites. For amplification of the PEX2 RING finger domain, 5′-CATGCCATGGGGCATGACTTGCCCTATTTGC-3′ and 5′-CCGCTCGAGTCATTTGCCACTTGAAAC-3′ were used to PCR-amplify PEX2RF with the *pfu* turbo enzyme (Stratagene, La Jolla, CA) from Arabidopsis total cDNA from light-grown seedlings. The product was cloned into *Nco*I and *Xho*I sites of the bacterial pET28a+ expression vector (Novagen, Madison, WI) to generate pHU010. Insert was confirmed by sequencing. Recombinant PEX2RF fused to 6xHis in the pET28a+ vector was expressed in bacteria and purified with nickel nitrilotriacetic acid agarose (Qiagen, Valencia, CA) according to the manufacturer’s protocol.

Constructs pHU011 and pHU012 were made by cloning the coding region of PEX2 RING finger and HY5, which had been amplified using the following PCR primer sets: 5′-GCGCAGGAGCTCATGACGCCGTCTACGCCTGC-3′ and 5′-GACTAGTTCATTTGCCACTTGAAACACCTTC-3′ for PEX2RF with *Sac* I and *Spe* I sites (restriction sites are underlined); and 5′-GCGCAGGAGCTCATGCAGGAACAAGCGACTAGCTCTTTAGC-3′ and 5′-CATGACCGTCGACAAAAGGCTTGCATCAGCATTAGAAC-3′ for HY5 (At5g11260) with *Sac* I and *Sal* I sites (restriction sites underlined). Restriction enzyme-digested PCR product was cloned at the *Sal* I and *Sac* I sites of pSY735 to generate pHU011 and *Sac* I and *Spe* I sites to generate pHU012. Both these constructs were verified by sequencing and subsequently digested with *Hind* III and subcloned into binary vector pZP221 for generating BiFC constructs pHU014 and pHU015. All constructs were confirmed by sequencing.

Vectors used in this study are described in Table S2.

### Antibody production

Polyclonal antibody was raised in rabbit against the PEX2 RING finger domain (aa 275–333) that had been purified to homogeneity from *E. coli* cells expressing the PEX2RF. ImmunoPure (Protein A) IgG Purification Kit (Thermo Fisher Scientific, Rockford, IL) was used to isolate IgG from the rabbit sera according to the manufacturer’s instructions. Purified IgG was desalted using Zeba desalt column using phosphate buffer (pH 7.2). A 1∶500 dilution of the desalted IgG fraction was used for all subsequent immunoblot assays.

### Yeast two-hybrid analysis

Full-length HY5, PEX2, ted3, and PEX2RF were restriction cloned into pGBKT7 (PEX2/ted3/PEX2RF) and pGADT7 (HY5) plasmids of the GAL4 Y2H system (Clontech, Mountainview, CA), using the method as previously described [17]. The yeast strain Y190 was transformed with the respective constructs and transformants selected on minimal media lacking leucine and tryptophan (–LW). Interactions were assessed by growing transformants in liquid culture at 30°C and spotting serial dilutions on –LW, –ALWH and –ALWH+25 mM 3-AT media. Plates were imaged after 2d of growth at 30°C. Immunoblotting of yeast extracts was carried out as previously described [17].

### Immunoblot analysis

Plant tissues (as indicated in the text) were ground to fine powder with liquid N_2_ and resuspended in 200 µl of buffer (400 mM sucrose, 50 mM Tris-HCl pH 7.5, 2.5 mM EDTA, 10 mM PMSF). Total extract was cleared by centrifugation and supernatant was mixed with 5x Lamelli buffer and resolved in a PAGE (polyacrylamide gel electrophoresis). Resolved protein was then transferred to PVDF membrane and blocked with 5% milk and 0.5% Tween-20 for 2 hr at room temperature and subsequently incubated with 1∶500 dilution of α-PEX2RF (Covance, Princeton, NJ), 1∶200 dilution of α-HY5 (Xing Wang Deng lab), or 1∶20,000 dilution of α-GFP (Abcam) overnight at 4°C. 1∶20,000 goat anti-rabbit IgG (Thermo Fisher Scientific, Rockford, IL) was used as the secondary antibody. The PVDF membrane was washed four times with 1X TBST for 10 min each time before the signals were visualized with SuperSignal West Dura Extended duration substrate (Thermo Fisher Scientific, Rockford, IL).

### Transient protein expression assays

*Agrobacterium tumefaciens* (strain GV3101) were transformed with the BiFC constructs and transformants were selected with 50 µg/ml kanamycin and 30 µg/ml gentamycin. Overnight bacterial cultures (28°C) of GV3101 containing the plasmid of interest was harvested by centrifugation, washed in water and resuspended in induction medium. Leaf infiltration was done as previously described [19]. Infiltrated plants were grown for 2 to 3 d in growth chambers before the leaf epidermal cells were examined for BiFC with epifluorescence or confocal microscopy.

## Results

### Overexpression of PEX2’s RING finger domain partially rescues det1

**Figure 1:**
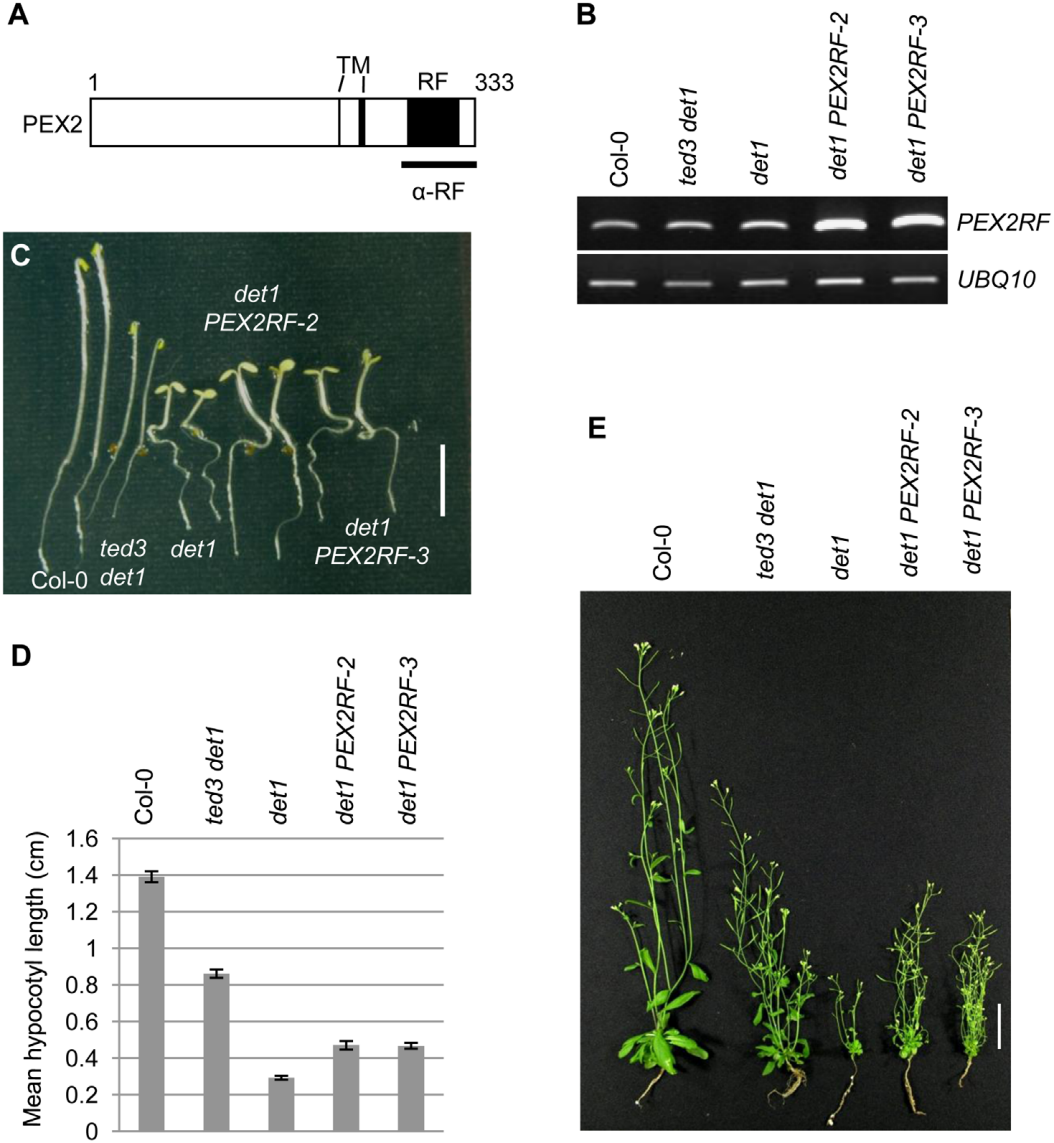
Overexpression of a peptide that contains the PEX2 RING domain suppresses *det1*. (A) Schematic of the Arabidopsis PEX2 protein, showing positions of the transmembrane (TM) and RING finger (RF) domains, and the region (indicated by horizontal bar) used as PEX2RF and as antigen for antibody generation. (B) RT-PCR analyses of the *PEX2RF* transcript in two transgenic lines (lines2 & 3) overexpressing PEX2RF in the *det1* background. *UBQ10* is the internal control. (C) Phenotype of 4d dark-grown seedlings grown on 0.5X MS supplemented with 0.5% sucrose. Scale bar = 0.5 cm. Two seedlings are shown for each genotype. (D) Hypocotyl length measurements of 4d dark-grown seedlings shown in (C). n>30 for each genotype. Student *t*-test, P<0.0001 for all lines vs. *det1*. Error bars indicate s.e.m. (E) Four-week plants. Scale bar = 3 cm.

To test the hypothesis that *ted3* creates a small peptide containing PEX2’s RING finger domain, which can translocate to the nucleus to partially compensate for the loss of a functional DET1, we first tested whether this RING domain is able to rescue the mutant phenotypes of *det1*. To this end, the *det1-1* mutants were transformed with a construct containing the RING finger (RF) domain of PEX2 (aa 275 to 333, Figure 1A) under the control of the 35S constitutive promoter. After RT-PCR analysis, two transgenic lines showing increases in the expression of *PEX2RF* mRNA compared with the *det1* control were selected for further analysis (Figure 1B). Dark-grown *det1* seedlings had short hypocotyls and opened cotyledons, whereas transgenic seedlings overexpressing PEX2RF had longer hypocotyls (Figure 1C). Quantification of the hypocotyl lengths of the transgenic seedlings proved this longer-hypocotyl phenotype to be significant (P<0.0001; Figure 1D). Further, adult transgenic plants were on average two times taller than *det1-1* although smaller than *ted3 det1-1* (Figure 1E). These results suggested that the seedling and adult phenotypes of *det1* can be partially suppressed by overexpression of PEX2’s RING finger domain.

### PEX2 RING-GFP localizes to the nucleus

**Figure 2:**
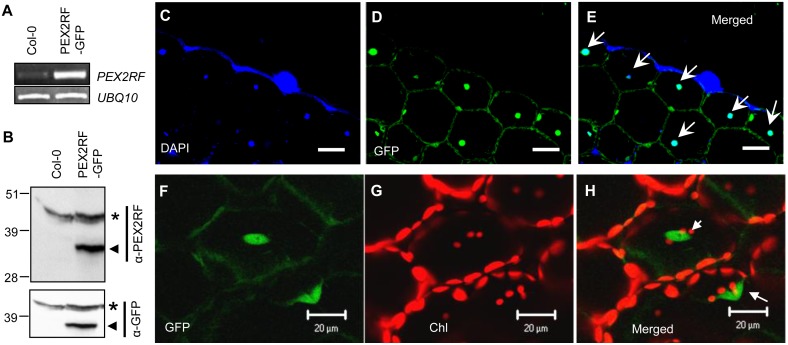
Nuclear localization of PEX2RF-GFP in transgenic plants. (A) RT-PCR analysis of *PEX2RF* mRNA and the *UBQ10* control in Col-0 and 35S::PEX2RF-GFP lines. (B) Immunoblot analyses of proteins from Col-0 and PEX2RF-GFP-expressing plants, using α–PEX2RF and α–GFP antibodies respectively. Asterisks indicate cross-reacting bands, and arrowheads point to the PEX2RF-GFP fusion protein. Numbers on the left indicate molecular weight markers in kDa. (C–H) Confocal images of transgenic plants from hypocotyl cells of 10d seedlings (C–E) and leaf mesophyll cells of two-week plants (F–H). DAPI stains the nucleus, green signals are from PEX2RF-GFP, and red signals are from chlorophyll autofluorescence. Arrows in the merged images indicate the nucleus. Scale bars = 10 µm in (C–E) and  = 20 µm in (F–H).

To determine whether the RING domain of PEX2 is capable of entering the nucleus, we generated a construct that expressed the PEX2RF-containing peptide (aa 275^Val->Met^ to 333) and fused it in-fame with a C-terminal green fluorescent protein (GFP). After generating transgenic lines expressing 35S::PEX2RF-GFP, semi quantitative RT-PCR analysis was performed to check for gene overexpression (Figure 2A). We also checked the presence of the PEX2RF-GFP protein with immunoblots, using a polyclonal antibody generated against PEX2’s RING domain (see Methods). This antibody detected the presence of overexpressed PEX2RF-GFP protein in plants (Figure 2B) and the overexpressed MBP-PEX2RF protein in yeast cells (Figure S1), but it failed to detect the hypothetical endogenous small peptide that contains PEX2RF in *ted3 det1*.

Transgenic plants expressing PEX2RF-GFP were subjected to confocal laser-scanning microscopy. Besides some localization in the cytosol, PEX2RF-GFP was primarily found in the nucleus in seedling hypocotyl (Figure 2C–2E) and leaf mesophyll cells (Figure 2F–2H). The presence of PEX2 RING domain in the nucleus and PEX2RF’s ability to partially suppress the *det1* phenotypes together suggested that this small peptide may be able to function in the nucleus to play a positive role in skotomorphogenesis, i.e. etiolation in the dark.

### PEX2RF interacts with HY5 in the nucleus

**Figure 3:**
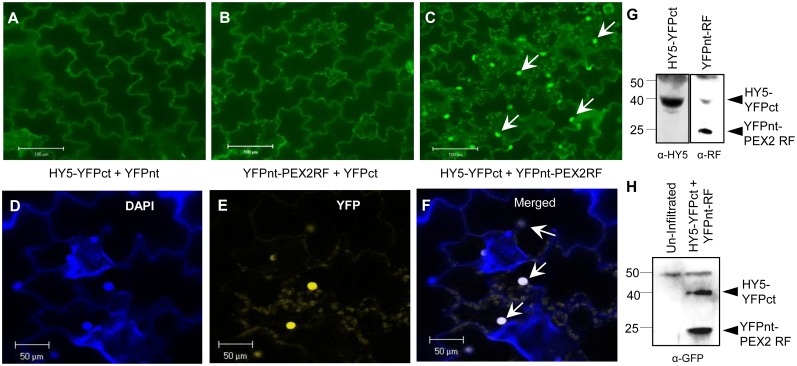
PEX2RF and HY5 interact in the nucleus. (A–C) Epifluorescence micrographs of tobacco leaf epidermal cells infiltrated with the indicated gene constructs. Strong YFP signals (BiFC) in the nucleus, as indicated by arrows in (C), were observed only when HY5-YFPct and YFPnt-PEX2RF were co-expressed. Scale bars = 100 µm. (D–F) Confocal micrographs of tobacco leaf epidermal cells co-infiltrated with HY5-YFPct and YFPnt-PEX2RF constructs. DAPI stains the nucleus (D), and BiFC signals are indicated by YFP fluorescence (E). Arrows in the merged image (F) indicate the overlaps of DAPI and BiFC. Scale bars = 50 µm. (G–H) Immunoblot analyses showing expression of HY5-YFPct and YFPnt-PEX2RF proteins in tobacco tissue. In (G), tissues were from plants shown in (A) and (B) respectively and α-HY5 (left) and α-RF (right) antibodies were used. In (H), tissue was from plant shown in (C), and α-GFP was used. Molecular weight markers in kDa are shown to the left of the blots.

Since HY5 is a key nuclear regulator of photomorphogenesis, we hypothesized that the nuclear localized PEX2RF may have an effect on HY5’s function. For example, it may physically interact with HY5 and allosterically modify its activity or stability. To determine whether PEX2’s RING finger domain and HY5 physically interact, we performed a Bimolecular Fluorescence Complementation (BiFC) assay [20] using tobacco (*Nicotiana tabacum*) plants. HY5 and PEX2RF were fused to the C- and N-terminal halves of YFP respectively to generate HY5-YFPct and YFPnt-PEX2RF. Epifluorescence and confocal microscopy analyses of infiltrated tobacco leaves revealed strong YFP complementation signals (BiFC) only when both proteins were expressed, and these YFP signals were enriched in the nucleus labeled by DAPI (Figure 3). These results confirmed that HY5 and PEX2RF were able to interact in the nucleus, where HY5 normally performs its function.

**Figure 4:**
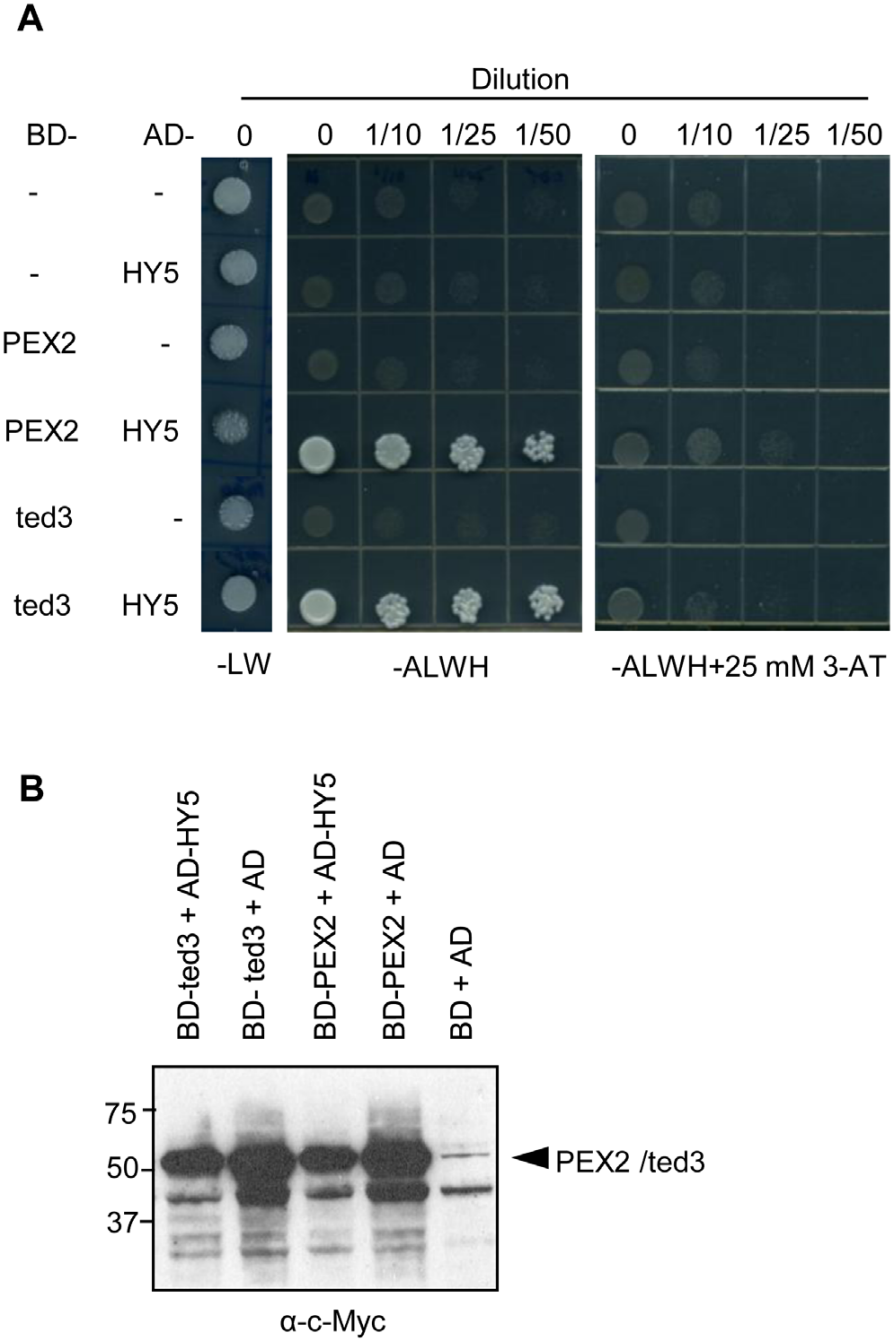
PEX2 and HY5 interact in yeast two-hybrid assays. (A) Yeast two-hybrid assays to show interaction between PEX2 and HY5. Yeast transformants containing the indicated GAL4 DNA binding domain (BD) and GAL4 activation domain (AD) fusion constructs were grown overnight in liquid culture and spotted on selection media plates (lacking leucine and tryptophan; –LW) and interaction media plates (lacking adenine, leucine, tryptophan and histidine; –ALWH, or –ALWH supplemented with 25 mM 3-amino-1,2,4-triazole; –ALWH+25 mM 3-AT). Growth on –ALWH and –ALWH+25 mM 3-AT media indicates protein interaction. (B) Immunoblot analysis of BD fusion constructs. Proteins extracted from transformed yeast cells shown in (A) were subjected to immunoblotting using α-c-Myc antibody. Numbers on the left of the blot indicate protein molecular weight markers in kDa.

We also employed yeast two-hybrid assays to test the interaction between PEX2RF and HY5 by fusing HY5 into the prey vector and PEX2/ted3/PEX2RF into the bait vector (see Methods). However, constructs containing PEX2RF autoactivated (Figure S2), so we focused on PEX2 and ted3 (i.e. PEX2 containing the 275^Val->Met^ substitution) instead. Both PEX2 and ted3 proteins were able to interact with HY5 (Figure 4), supporting the conclusion that PEX2 can physically interact with HY5 and that this interaction is likely mediated by the RING domain of PEX2.

### HY5’s function in photomorphogenesis is compromised in ted3 det1

**Figure 5:**
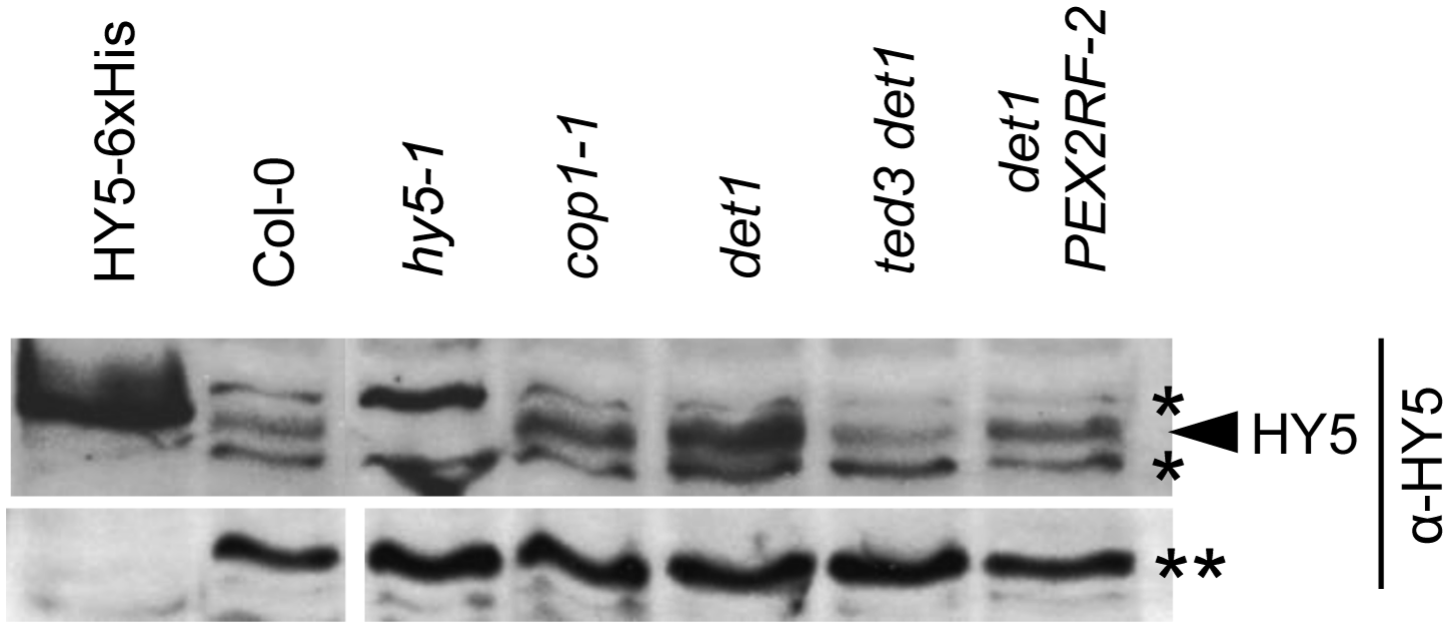
Immunoblot analysis of the HY5 protein in various genetic backgrounds. Proteins were extracted from 4d dark-grown seedlings exposed to 1 hr white light and detected with the α–HY5 antibody. Purified HY5–6xHis from our previous study [11] was used as a control. Asterisks indicate non-specific bands. A cross-reacting band indicated by a double asterisk served as the loading control.

To explore the possible physiological relevance of this protein-protein interaction between HY5 and PEX2RF, we checked the abundance of HY5 in dark-grown seedlings in various genetic backgrounds. HY5 is the target for degradation by the COP-DET1 complexes in the dark; lack of or significant reduction of the level of this protein leads to long hypocotyls in light-grown seedlings [6]. Conversely, in mutants of the COP-DET1 complexes such as *det1* and *cop1*, HY5 is stabilized and thus dark-grown seedlings display a de-etiolated phenotype by having short hypocotyls [7]. Similar to what had been shown previously, HY5 showed higher accumulation in *cop1-1* and *det1-1* mutants when compared with wild-type Col-0, whereas this higher accumulation was reduced in *ted3 det1-1* and to lesser degrees, in *det1* mutant overexpressing PEX2RF (Figure 5). These results led us to speculate that PEX2’s RING finger domain in the nucleus may be involved in inactivation and/or turnover of HY5 directly or indirectly.

**Figure 6:**
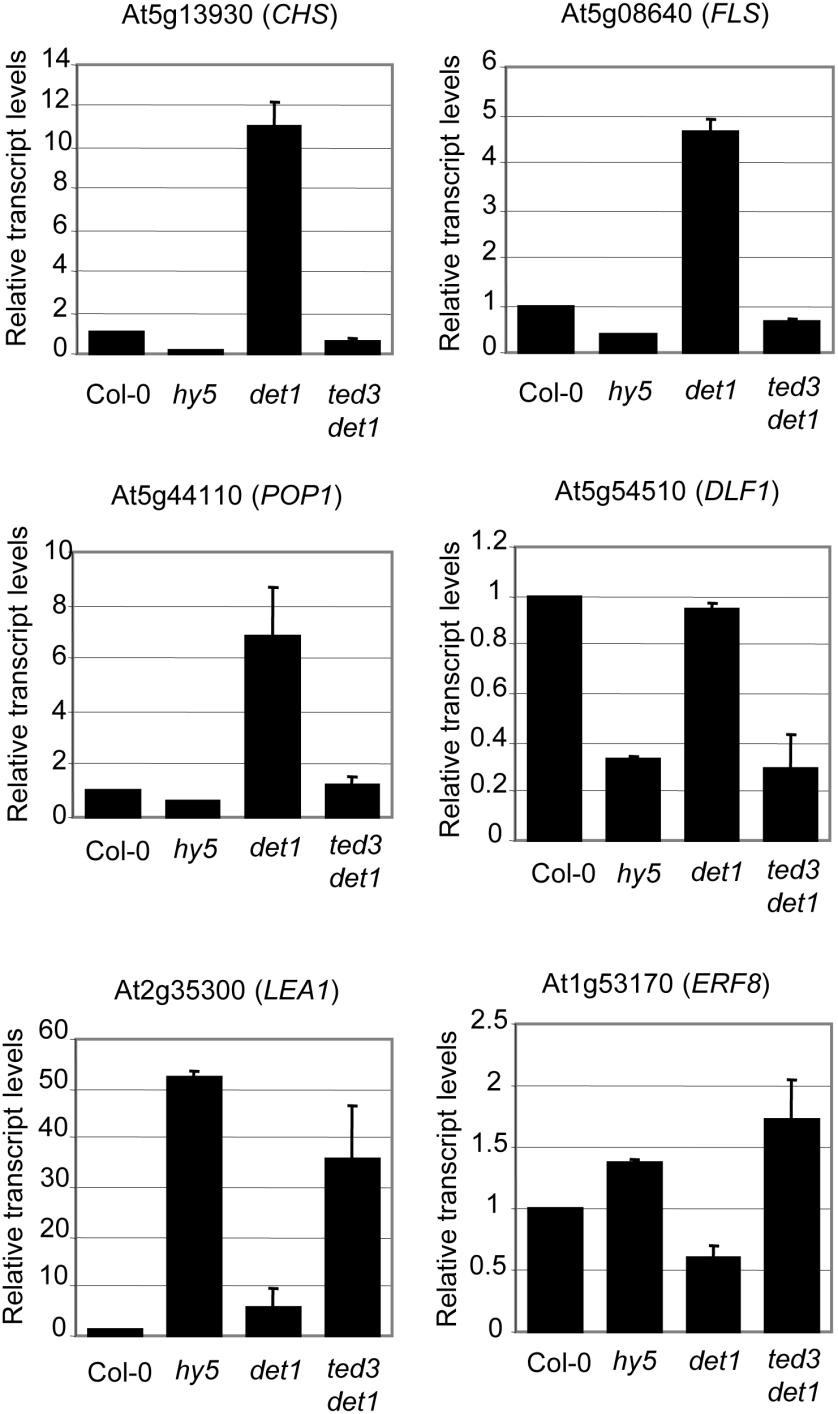
qRT-PCR analysis of the expression of some of HY5’s target genes. RNA was extracted from 4d dark-grown seedlings in different genetic backgrounds. Three biological replicates of qRT reaction were performed for individual primer sets. The transcript level of each gene in the mutant is represented as arbitrary unit relative to the transcript level of the same gene in the wild-type plant, which was set to 1.0. The transcript level (relative expression) is the ratio between the transcript abundance of the studied gene and the transcript abundance of *UBQ10*. Values correspond to the mean and s.d. of three biological replicates. The experiments were repeated twice with consistent results.

Given the significant reduction of the level of HY5 in *ted3 det1*, we reasoned that the downstream events regulated by HY5 may also be reversed in this suppressor to revert *det1*’s phenotype. To test this, we selected six light-regulated genes that are known to be direct targets of HY5, and performed expression profiling by quantitative real-time PCR of these genes in Col-0, *hy5-1*, *det1-1*, and *ted3 det1-1*. Those positively regulated by HY5 included genes that encode chalcone synthase (CHS) and flavonol synthase (FLS), which are involved in anthocyanin/flavonoid biosynthesis [21], the ABC transporter POP1 (P-loop containing nucleoside triphosphate hydrolases superfamily protein) [22], and the auxin signal transduction component Dwarf in Light1 (*DFL1*) [23]. The two genes negatively regulated by HY5 encoded the late embryogenesis abundant protein LEA1 and ethylene response factor ERF8 [22]. As expected, transcript levels of *CHS, FLS, POP1, and DLF1* decreased in *hy5-1* but increased in *det1.*, For the genes negatively regulated by HY5, ERF8 was up-regulated in *hy5-1* and down-regulated in *det1*, whereas *LEA1* was up-regulated in both *hy5-1* and *det1* (Figure 6). In *ted3 det1*, the altered expression pattern shown in *det1* was reversed for five of the six genes to levels similar to those in the *hy5* mutant (Figure 6), supporting the notion that HY5 activity in *ted3 det1* is compromised, which may be a major cause for the partial reversal of the *det1* phenotype.

## Discussion

COP/DET1/FUS are global repressors of light-regulated development, functioning in the proteolysis of positive regulators of photomorphogenesis in the nucleus [4], [5]. The identification of a dominant peroxisomal mutation that suppressed the de-etiolated phenotype of *det1* was intriguing [14]. One plausible explanation was that the Met created at position 275 in *ted3* initiated the translation of a small peptide that contains the C-terminal RING finger domain of PEX2. Alternatively, this Val-to-Met change renders the protein more susceptible to proteases, which cleave off the RING domain of PEX2 in the cytosol. The RING domain-containing peptide then translocates to the nucleus to substitute the function of DET1 in photomorphogenesis. We have not been able to unequivocally prove the above hypothesis in this study, as we could not detect the hypothetical small peptide derived from the Val-to-Met substitution in *ted3 det1* or *ted3* overexpressors. This is possibly due to insufficient avidity of the PEX2 antibody we generated and/or the low abundance/instability of this peptide. However, we have shown in this study that overexpression of a small peptide containing PEX2 RING domain in *det1* can indeed partially suppress the *det1* phenotype. Majority of the PEX2RF-GFP protein was seen in the nucleus, and only a small portion of the fusion protein was visible in the cytoplasm. We speculate that PEX2RF passively enters the nucleus due to its small size (∼6 kDa), although we do not rule out the possibility that it goes to the nucleus through active targeting or other mechanisms.

The RF domains of PEX2 and COP1 both belong to the C_3_HC_4_ type. When overexpressed in wild-type Arabidopsis plants, an N-terminal fragment of COP1 that contained both the RING finger and coiled-coil domains was found in the nucleus and conferred a dominant negative effect that mimicked the phenotype of *cop1*. The phenotype was believed to be caused by the interaction between this peptide and the endogenous COP1, which resulted in the interference with COP1’s normal function [24]. In our study, PEX2 RF alone was overexpressed in the mutant *det1* background and conferred phenotype opposite to that of the COP1 study. Our BiFC and yeast two-hybrid assays demonstrated the interaction between PEX2 and HY5. In addition, the accumulation of the HY5 protein in *det1* was reduced in *det1 PEX2RF* and *ted3 det1*. Furthermore, the altered expression of five out of the six analyzed HY5 target genes in *det1* was reversed in *ted3 det1*, prompting us to speculate that this reduced activity of HY5 was at least in part responsible for the suppression of *det1*. This is also consistent with a previous report, which showed that HY5 inactivation in *cop1* and *det1* mutants resulted in reversal of their dark-grown phenotypes [25]. How does ted3 reduce the level of HY5? Given that PEX2RF contains E3 ubiquitin ligase activity [17], ted3 may be directly involved in the degradation of HY5. *ted3* also partially suppressed *cop1* but not *det2*, a de-etiolated mutant deficient in an enzyme in brassinosteroid biosynthesis [14]. Therefore, *ted3* seems to have some specificity toward the COP/DET1-associated photomorphogenic pathway, which makes sense given that HY5 is a major target of the COP/DET1 proteolytic complexes. Finally, since HY5 is not the only target of DET1’s function, reducing HY5 activity may not be sufficient to completely rescue the *det1* mutant phenotypes.

Although we have not been able to prove this hypothesis, we predict that the partial suppression of *det1* by *ted3* is primarily due to the creation of a RING finger-containing peptide that replaces the function of DET1 in the nucleus, and not due to changes in peroxisomal function. Replacement of a Val, which is nonpolar, by the partially charged Met was shown to affect the function of proteins related in human diseases [26], [27], [28]. Similarly, substitution of Val by Met may distort the overall configuration of the cytoplasmic end of the PEX2 protein thus affecting the activity of the RING finger domain, resulting in a PEX2 protein with mildly reduced activity in peroxisome biogenesis.

RING-type E3 ligases mediate ubiquitination and are implicated in diverse developmental processes across kingdoms [29]. Our work supports the possibility that a gain-of-function mutation in a peroxisomal gene can have a marked effect on the function of a nuclear protein. Given the conservation of the RING finger domain among proteins in various genomes, it is interesting to speculate that some other RING domains when appropriately localized may also cause neomorphic phenotypes.

## Supporting Information

Figure S1 **Specificity of the PEX2RF antibody.** (A) SDS-PAGE gel showing induction of the expression of the fusion of maltose binding protein (MBP) and PEX2RF in bacterial protein lysates. U, Is and Ip stand for uninduced, soluble and pellet fractions, respectively. Protein expression constructs have been previously described in Kaur *et al*., 2013 [17]. Arrow and arrowhead point to MBP alone and MBP-PEX2RF respectively. (B) Immunoblot analysis of MBP-RF expression in induced bacterial protein lysates, as detected by the PEX2RF antibody. Numbers on the left of the blots indicate molecular weight markers in kDa.
(TIF)

Figure S2 **PEX2RF auto-activates in yeast two-hybrid assays.** (A) Yeast cells transformed with BD and AD constructs were spotted on selection (−LW) and interaction media (−ALWH and –ALWH+25 mM 3-AT). Strains containing BD-PEX2RF grow on interaction media even in the absence of HY5, indicating that the RF autoactivates. (B) Immunoblot analysis to detect the expression of BD-PEX2RF fusion proteins in yeast cells, using anti-c-Myc and anti-PEX2RF antibodies respectively.
(TIF)

Table S1 **Primers used in qRT-PCR.**
(PDF)

Table S2 **Vectors used in this study.**
(PDF)
