# Supplementary figures and images for: Ectopic Expression of the RING Domain of the Arabidopsis PEROXIN2 Protein Partially Suppresses the Phenotype of the Photomorphogenic Mutant De-Etiolated1
Source: PLoS One. 2014 Sep 23;9(9):e108473. doi: 10.1371/journal.pone.0108473 (PMC4172754; doi:10.1371/journal.pone.0108473)

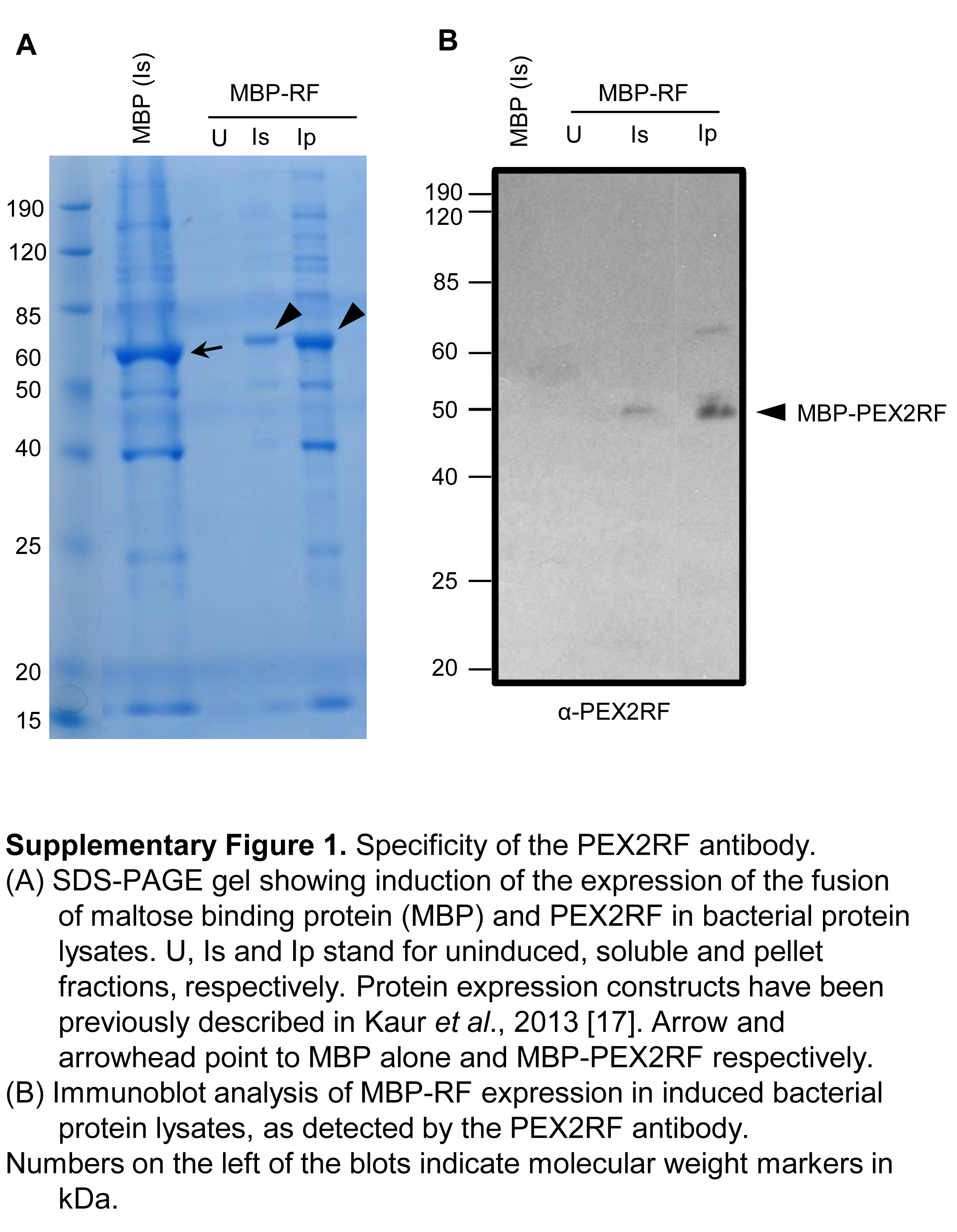

Supplement: Figure S1 — Specificity of the PEX2RF antibody. (A) SDS-PAGE gel showing induction of the expression of the fusion of maltose binding protein (MBP) and PEX2RF in bacterial protein lysates. U, Is and Ip stand for uninduced, soluble and pellet fractions, respectively. Protein expression constructs have been previously described in Kaur et al., 2013 [17]. Arrow and arrowhead point to MBP alone and MBP-PEX2RF respectively. (B) Immunoblot analysis of MBP-RF expression in induced bacterial protein lysates, as detected by the PEX2RF antibody. Numbers on the left of the blots indicate molecular weight markers in kDa. (TIF) [file pone.0108473.s001.tif]

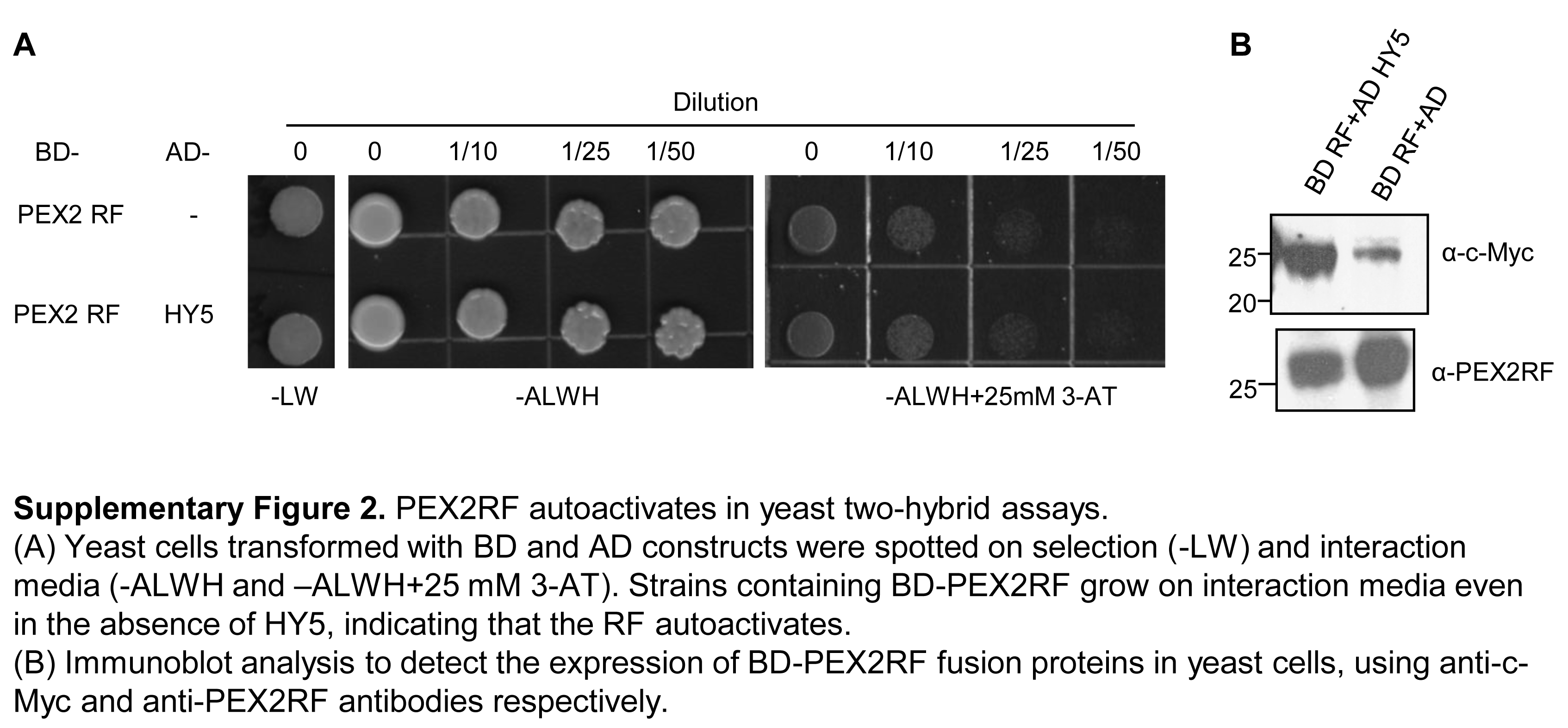

Supplement: Figure S2 — PEX2RF auto-activates in yeast two-hybrid assays. (A) Yeast cells transformed with BD and AD constructs were spotted on selection (−LW) and interaction media (−ALWH and –ALWH+25 mM 3-AT). Strains containing BD-PEX2RF grow on interaction media even in the absence of HY5, indicating that the RF autoactivates. (B) Immunoblot analysis to detect the expression of BD-PEX2RF fusion proteins in yeast cells, using anti-c-Myc and anti-PEX2RF antibodies respectively. (TIF) [file pone.0108473.s002.tif]
