# Supplementary material for: Ectopic Expression of the RING Domain of the Arabidopsis PEROXIN2 Protein Partially Suppresses the Phenotype of the Photomorphogenic Mutant De-Etiolated1
Source: PLoS One. 2014 Sep 23;9(9):e108473. doi: 10.1371/journal.pone.0108473 (PMC4172754; doi:10.1371/journal.pone.0108473)
Supplement: Table S1 — Primers used in qRT-PCR. (PDF) [file pone.0108473.s003.pdf]

Table S1. Primer used in qRT-PCR.

AGI, Arabidopsis Genome Initiative.

Gene	AGI Code	Primer Pairs
FLS	At5g08640	5'-ATCTAAGCGATCCCGACGAA-3'
		5'-CCCATTCTTCACTCGCTTTCA -3'
AP2/EREBP	At1g53170	5'-CAGCCGCGCGTGACTT-3'
		5'-CTACCAACGATAACACCGAAATTG-3'
DFL1	At5g54510	5'-AAACGACACGGGCTCGAA-3'
		5'-ACGACAGGCATGATATGTTTGAA-3'
POP1	At5g44110	5'-CGAGACATGGGCTAGTCATTTG-3'
		5'-CATCTAACTTGGCCGAGAGTTTC-3'
CHS	At5g13930	5'-AAGCGCATGTGCGACAAG-3'
		5'-TCCTCCGTCAGATGCATGTG-3'
LEA	At2g35300	5'-AACATCGGTGGCGCAAAG-3'
		5'-TTTTTGGTCCTTGCCATCGT-3'
UBQ-10	At2g36790	5'- TTTTCGCCGGCAAGCA-3'
		5'- TGGATTCCTTCTGGATATTGTAATCA-3'
RGL2	At3g03450	5'-ACCGAGTATCGTAACGGTGGTT-3'
		5'-AACCTATCGAGGAAGACGATTCC-3'
