# Supplementary material for: Ectopic Expression of the RING Domain of the Arabidopsis PEROXIN2 Protein Partially Suppresses the Phenotype of the Photomorphogenic Mutant De-Etiolated1
Source: PLoS One. 2014 Sep 23;9(9):e108473. doi: 10.1371/journal.pone.0108473 (PMC4172754; doi:10.1371/journal.pone.0108473)
Supplement: Table S2 — Vectors used in this study. (PDF) [file pone.0108473.s004.pdf]

**Table S2.** Vectors used in this study.

| Construct | Vector | Description |
|---|---|---|
| *HU010* | pET28a(+) | PEX2 RING finger cloned for C-term His-tag fusion. |
| *HU006* | pCHF3:GFP | PEX2 RING finger cloned for C-term GFP-Tag fusion. |
| *HU007* | pCAMBIA1300 | PEX2 RING finger cloned for overexpression. |
| *HU011* | pSY735 | pSY735-YFPct-HY5. |
| *HU012* | pSY736 | pSY736-YFPnt-PEX2RF. |
| *HU014* | pZP221 | For performing BifC with pSY735-YFPct-HY5. |
| *HU015* | pZP221 | For performing BifC with pSY736-YFPnt-PEX2RF. |
| | pGBKT7 | To generate BD-PEX2/ted3/RF fusion Y2H constructs |
| | pGADT7 | To generate AD-HY5 fusion Y2H construct |
